# A Review of the Advantages, Disadvantages and Limitations of Chemotaxis Assays for *Campylobacter* spp.

**DOI:** 10.3390/ijms23031576

**Published:** 2022-01-29

**Authors:** Bassam A. Elgamoudi, Victoria Korolik

**Affiliations:** 1 Institute for Glycomics, Griffith University, Gold Coast Campus, Southport, QLD 4222, Australia; b.elgamoudi@griffith.edu.au; 2 School of Pharmacy and Medical Science, Griffith University, Gold Coast Campus, Southport, QLD 4222, Australia

**Keywords:** chemotaxis, *Campylobacter jejuni*, chemoeffector screening, chemotaxis assays

## Abstract

Reproducible qualitative and quantitative assessment of bacterial chemotactic motility, particularly in response to chemorepellent effectors, is experimentally challenging. Here we compare several established chemotaxis assays currently used to investigate *Campylobacter jejuni* chemotaxis, with the aim of improving the correlation between different studies and establishing the best practices. We compare the methodologies of capillary, agar, and chamber-based assays, and discuss critical technical points, in terms of reproducibility, accuracy, and the advantages and limitations of each.

## 1. Introduction

Chemotaxis directed motility of intestinal bacteria such as *Campylobacter jejuni* enables the cells to move toward favourable conditions and away from hazardous ones and has been shown to be involved in colonisation and disease [1,2,3,4,5,6]. A number of assays have been developed to investigate bacterial chemotaxis [7,8,9], including the capillary and hard plug agar assays (HAP), which are extensively used to study bacterial chemotactic responses to chemoeffectors [10,11]. However, in many cases, the results of different studies lack consistency (particularly when applied to campylobacters) and reproducibility, in addition, they demonstrate excessive experimental variation, unsuitability for studying chemorepellents, and false positive responses [12,13,14,15,16,17]. Moreover, the measurements of migration by chemotaxis assays can be complicated due to the metabolic consumption of chemoeffectors, which may create a secondary gradient that the cells can sense. In order to circumvent these limitations, alternative chemotaxis assays have been developed to investigate the chemotactic behavior of *Campylobacter* spp., including a nutrient-depletion assay, t-HAP assay, tube-based assay, and μ-slide chemotaxis chamber. Here, we compare the complexity, scope of obtained information, observable qualitative impact and quantitative accuracy of these methods for the study of the responses of *C. jejuni* to chemoattractants and chemorepellents, with respect to each other and other published methodologies.

## 2. Agar Plug-Based Assays

Agar plug-based assays were initially introduced for studying chemotaxis of *Escherichia coli* [18,19]. In these assays, a plug of hard agar containing an attractant, or a repellent is placed in a petri dish containing soft agar, at a low enough concentration so that the bacteria can swim, mixed with bacterial cells concentrated enough to be visibly turbid. This assay has been widely adapted and used for other bacteria such as *Shewanella oneidensis, Helicobacter pylori* [20], and *Pseudomonas* spp. [21]. The advantage of this assay is that it is easy to set up, and a response can usually be seen by eye in about 30 min.

### 2.1. Hard Plug Agar Assay (HAP)

The hard agar plug (HAP) assay, as described by Hugdahl et al. [16], has been extensively used to study changes in campylobacterial chemotactic motility. This is a simple assay where plugs of agar, containing chemoeffectors, are placed in semisolid agar (0.35% agar) containing a dense suspension of bacterial cells (~10^9^ cfu/mL). Cells swim in the soft agar through the concentration gradient toward a chemoeffector in the HAP. A visually observable cloudy zone condenses around the HAP if it contains an attractant (positive chemotaxis), or a zone clearing appears around the HAP if contains a repellent (negative chemotaxis). For quantitation, cloudy zones of bacterial cell accumulation around a plug or zones of bacterial clearing, are measured by a ruler from the edge of the plug to the edge of the zone and compared to the control plug. However, the catabolised ligands and their metabolic products could interfere with the accurate measurement of the chemoresponses, as the accumulation of bacterial cells around plugs containing such chemoattractant could create a secondary gradient that the bacteria can sense. For example, catabolised ligand L-serine can be used as a carbon and energy source by *C. jejuni* [22,23]. Serine is converted to pyruvate which is also a chemoattractant for *C. jejuni* [24,25] and induces bacterial growth. In addition, the measurement of the extent of the dense or cleared zones around the HAPs is dependent on the judgement of the operator and can vary from assay to assay and study to study.

A number of modified HAP assays have been trialed in order to qualitatively and quantitatively assess the changes in campylobacterial chemotactic behaviour [13,14,26,27,28,29]. For instance, Du, et al. [30] used a modified HAP assay to investigate the role of *Cj0371* in chemotaxis. This gene was proposed to be associated with virulence, and a mutant of *C. jejuni* Δ*cj0371* had a stronger chemoresponse to malic, ketoglutaric and succinic acids. However, these organic acids are metabolised by the organism, and when the cells are incubated for 6 h, metabolic byproducts are likely to create secondary gradients that the bacteria can also sense. Another modified assay, by Hazeleger, et al. [31], employed filter paper discs instead of agar plugs in a study of *C. jejuni* behaviour at environmental temperatures. Cell suspensions were prepared as described by Hugdahl et al. [16], mixed with 0.4% PBS-agar (Phosphate Buffered Saline) at 45 °C, poured into 9-cm-diameter Petri dishes, and filter paper discs saturated with 30 μL of 1 M of sodium malate, sodium formate, or sodium pyruvate (pH 7) were placed on the cell-agar mixture. Plates were then incubated aerobically or microaerobically at 4, 20, or 40 °C and examined after 0.5 h to 16 h. The results suggested that *C. jejuni* strains respond to formate at all tested temperatures, and the appearance of turbid zones was due to the cell migration toward the substrate. In another study [28], the chemoresponses of *C. jejuni* isolates toward a variety of amino acids, sodium salts and sugars were similarly assayed. Positive chemoresponses were reported toward phenylalanine, l-tryptophan, cysteine and citrate, while the capillary method, which we will discuss later, was used to confirm the data, showed chemoresponse to citrate only. The discrepancy in the chemotaxis assays outcomes had most likely resulted from the use of other than neutral pH of amino acid solutions as well as the extended incubation time that may have allowed for bacterial growth (16 h). In contrast, a similar study by Vegge et al. reported chemoresponses of *C. jejuni* NCTC11168 to l-aspartate, cysteine, fumarate, l-glutamate, α-ketoglutamate, pyruvate, l-serine, succinate, formate, d-lactate and malate a 100 mM effector concentration as chemoattractants after 4 h of incubation at 37 °C [12,13,14,15,16,17]. In addition, the same study identified bile components such as cholic, deoxycholic, taurocholic, and glycocholic acids as chemorepellents. Interestingly, using the same methodology, Li, et al. [12] could not detect any *C. jejuni* chemoresponses toward cysteine, l-serine, pyruvate and swine mucin, and in contrast with Hugdahl et al. [16] and Vegge et al. [13], found sodium deoxycholate, bovine bile and human bile to be chemoattractants.

In an attempt to address such discrepancies and to introduce a quantitative component to the assay, Elgamoudi et al. [26] introduced a new modification to a HAP assay, a t-HAP assay. This modified method enables the user to test responses to chemoattractants and chemorepellents and includes triphenyl tetrazolium chloride (TTC) as a cell viability indicator. Colourless TTC is added to HAP plugs and is converted to red coloured 1,3,5-*triphenylformazan* (TPF) by metabolically active respiring bacterial cells, without any effect on growth. The chemoresponses can be detected following a relatively short incubation time (10 min to 3 h), where a visual chemoresponse can be observed as a turbid red halo appearing around the plug containing a potential attractant. In agreement with Hugdahl et al. [16] and Vegge et al. [13], t-HAP assay showed that *C. jejuni* NCTC11168-O responds to L-aspartate, fumarate, glutamate, α-ketoglutarate, L-serine, fucose, cysteine, proline and malate as chemoattractants. Additionally, this method enables quantitation of the chemoresponse by colorimetry, where the area (~3 cm^2^) around the red-halo zone around the plug can be collected, transferred into 4 mL of PBS, vortexed until the agar is thoroughly particulated, filtered, and the absorption is measured at 600 nm. This modified assay is also suitable for identifying the responses to the chemorepellents (chemorepellent assay), by mixing a known chemoattractant (i.e., L-serine) with a potential chemorepellent in a single HAP plug. This allows visualisation of a repellent effect as a reduction in the response to a control chemoattractant, which is present in the mixture at the same concentration as the control. t-HAP assay was able to confirm that glucosamine, lysine, phenylalanine, l-tryptophan, tyrosine, and thiamine induced a chemorepellent effect in *C. jejuni* 11168-O [26]. Another advantage of the t-HAP assay is that it enables differentiation between catabolised and non-catabolised chemoeffectors, such as isoleucine, as the t-HAP chemorepellent assay offers both visual and quantitative measurements by colourimetry and viable count [26]. Subsequently, Khan, et al. [32] used the t-HAP assay to identify additional chemoattractants including leucine, valine, α-amino-N-valeric acid, 4-methylisoleucine, β-methylnorleucine, 3-methylisoleucine, alanine, and phenylalanine.

While technically undemanding, most HAP-based assays do have a range of limitations and disadvantages, described in Table 1, as in both qualitative and quantitative form, these assays rarely produce results in a consistent and reproducible manner [20,33]. For instance, most HAP-based assays are semi-qualitative, with the exception of t-HAP, and while this assay provides clear, visible and easy to interpret results, it does not allow the measurement of parameters such as threshold concentration or response strength, making dose-dependent and comparative analysis of responses to different stimuli by different strains unreliable. In addition, there is a great amount of inconsistencies in the published agar-based methods in regard to the use of controls for chemosensory motility [20,33]. Of greatest concern is the use of buffers, such as PBS, as sole controls, instead of non-motile and non-chemotactic mutant strains, such as non-motile (Δ*flaAB*) or non-chemotactic (Δ*cheY*) strains, which have now been shown to be critical for any chemotaxis assay in order to validate the results and to avoid false positives [20,33].

### 2.2. Tube-Based Chemotaxis Assays

This assay was first described by Reuter et al. [27] for characterisation of the energy taxis genes, *cj1190c* (*cetA*), *cj1189c* (*cetB*) and *cj1110c* (*cetZ*) in *C. jejuni.* The assay was adapted by Dwivedi, et al. [27] to investigate the fucose chemotaxis in *C. jejuni.* In this assay, bacterial cells in 0.4% PBS-agar are transferred to the bottom of a 2 mL Eppendorf tube, allowed to solidify and then overlaid with 1 mL of 0.4% PBS-agar. A filter paper soaked with 50 µL of a chemoeffector (i.e., L-fucose, L-serine) is placed on top of the agar and incubated under microaerobic conditions for 72 h at 37 °C. Bacterial cells that migrate through the upper layer of PBS-agar towards a chemoeffector in the filter paper can be visualised by adding TTC. As TTC changes colour to red in the presence of metabolic activity, the chemoattractant effect can be observed by formation of a red ring of bacterial cells on the top of the tube, visible after 3–4 h of additional incubation [36,40]. The additional advantage of this assay is that the bacteria accumulated in the top layer of the agar can be collected and quantitated by viable count allowing the collection of both qualitative and quantitative data. Unfortunately, this assay is not suitable for the assessment of chemorepellents and the 72 h incubation time could lead to an increase in cell number due to growth and can thus affect the measurement of chemotactic activity (Table 1). The controls in this assay became even more difficult to design, as different metabolites affect the increase in the bacterial numbers, due to growth, differently.

### 2.3. Nutrient-Depletion Assay

The nutrient-depletion assay has been developed for the quantitative assessment of both chemoattractants and chemorepellents [35,41]. Briefly, 0.5% agar (in H_2_O without any nutrients) is poured into a petri dish and plugs of 6 mm are removed and then replaced with 0.5% agar with 2 mM of a chemoeffector. The plates are overlaid with 0.1% agar in H_2_O and left for 2 h to allow for the diffusion of chemoeffectors to create a chemical gradient. *C. jejuni* cells (~10^8^–10^9^ cfu/mL) in a 100 μL of bacterial suspension are inoculated in the centre of the petri dish and incubated at 37 °C for 4 h to allow chemotactic migration of the cells. To determine the number of viable bacteria associated with each plug, a 5 mm area around and including each plug is removed and quantitated by viable count. This assay was used to identify ligands for a number of *C. jejuni* chemoreceptors. Rahman, et al. [35] demonstrated the multi-ligand binding of chemoreceptor Tlp3 (CcmL) which is able to sense isoleucine, purine, malic acid and fumaric acid as chemoattractants and lysine, glucosamine, succinic acid, arginine and thiamine as chemorepellents. Day, et al. [34] used this assay to identify the first galactose chemoreceptor in *C. jejuni*, while Elgamoudi, et al. [39] showed that *C. jejuni* chemoreceptor Ttlp10 can sense different classes of ligands, amino acids and glycans as either chemoattractants or chemorepellents. The advantage of this assay is that each plate contains two controls, a positive (WT strain) and a negative (*flaA*^−^/*flaB*^−^) control, and importantly, this assay can test the chemoeffectors at a biologically relevant concentration (2 µM instead of 100 µM) without the possibility of bacterial growth. The key limitation of this method is the requirement for skillful manual handling by an experienced operator as the assay is highly sensitive to vibration.

## 3. Capillary Assays

The capillary chemotaxis assay had been considered as a “gold standard” for many years and was the most commonly used method to assess bacterial chemotaxis in which errors due to metabolic activity and growth can be minimized [18,42,43]. In this assay, the chemotaxis is monitored by measuring the number of bacterial cells entering a capillary tube over a period of hours in the presence or absence of chemoeffectors. In brief, a capillary tube, 1 µL disposable micropipette (3 cm long with an internal diameter of 0.2 mm), containing a solution of an attractant, and sealed at one end, is inserted into a bacterial suspension. A spatial gradient is formed by the diffusion of the attractant/from the tip of the capillary tube. After incubation for 30–60 min, the capillary is removed, and the sealed end is broken off over a test tube containing tryptone broth to be ready for a viable count. For positive chemotaxis, the number of cells accumulated inside a capillary containing attractant solution is measured. For negative chemotaxis, the repellent effector in the capillary decreases the number of cells as opposed to the cell numbers accumulated due to random motion. Driven by the level of handling difficulty, expertise required and low reproducibility, particularly in the assessment of chemorepellents, a number of modifications were introduced over time.

One capillary based assay had been modified to enable the quantitative measurement of bacterial chemoresponses for *Pseudomonas* spp. [44,45,46,47], *H. pylori* [48] and *Campylobacter* spp. [24,37]. Briefly*, C. jejuni* cells are harvested into PBS buffer to OD_600_ of 0.5. A 100 μL of a solution containing 100 mM of a chemoeffector is aspirated through a stainless-steel needle (0.25 mm diameter × 20 mm long) into a 1 mL tuberculin syringe. A 100 μL of the bacterial suspension is then drawn into a 200 μL disposable pipette tip, which is then sealed at one end. The needle-syringe system is fitted to a pipette tip in such a way that most of the needle is immersed into the bacterial suspension and incubated horizontally for 1 h allowing the cells to migrate toward an effector. Bacterial cells migrated into the syringe are enumerated by viable count. To test the response to repellents, bacterial suspension can be mixed with a repellent and the bacteria that enter the syringe, which in this instance contains only the buffer, are allowed to escape the repellent. The accuracy of the repellent effect can be compromised due to the unknown level of lethality that the repellent can induce in the viable cells. The effect is quantified as the Relative Chemotaxis Ratio (RC). RC is calculated as the ratio between the numbers of bacteria entering the test needle-syringes and those in the control (PBS buffer only) needle-syringes. The ratio is sometimes given without actual cell numbers, and if the number of bacterial cells is low (RC of three, when considering cell numbers of 10 to 100 cells vs. the same ratio with 10^6^–10^8^ cells), this can lead to a misleading or erroneous conclusion, particularly if appropriate non-chemotactic controls are not used. This method had been used by Lübke, et al. [24], to characterise *C. jejuni* Tlp12, which was found to be involved in glutamate and pyruvate chemotaxis. They also found that *C. jejuni* has chemoresponses to L-aspartate, glutamine, lactate, L-serine, and succinate. In agreement with the previous finding, Chandrashekhar, et al. [37,49] found that *C. jejuni* has chemoresponses to L-aspartate, glutamine, L-serine, formate, succinate, glutamate, propionate, and pyruvate. Interestingly, iron and phosphate showed an effect on *C. jejuni* chemoresponses [49]. While the capillary tube was not used by Huang, et al. [50], they constructed a sophisticated phase contrast microscopy system to observe *H. pylori* chemoresponses to urea in real-time by inserting a micropipette with bacteria into a stable gradient of the effector within the field of observation. They found that *H. pylori* chemoreceptor, TlpB, can detect urea at a nanomolar concentration which permits *H. pylori* cells to direct their movement toward the gastric epithelium in order to colonise the host. While this method can demonstrate bacterial chemoresponses in real-time, it is difficult to set up, lacks quantitation, requires specialised equipment, and requires an extremely skilled handler.

Overall, a critical limitation of capillary assays is the requirement of skillful manual handling of the capillary tube which greatly affects the reproducibility and accuracy of the results. Another important point to consider for all capillary assays is the effector concentration, as most of tested concentrations range from 100 mM to 1 M and are likely to be too high to be biologically relevant. This is particularly important as some amino acids have been shown to act as attractants at low concentrations but as repellents at higher concentrations [51].

## 4. Slide-Based Chemotaxis Assay

Recently developed microscopic tracking systems can provide a powerful alternative tool to assess bacterial motility and chemotaxis [52,53,54]. This system allows for a more standardised approach to tracking a group of cells or a single cell through microscopy and time–lapse images measure many features of bacterial motility such as cell migration, velocity, and navigational behavior. A good example is an assay using an agarose-in-plug bridge method, employed to study chemotaxis in many organisms, such as Archaeon *Halobacterium salinarum*, *Escherichia* *coli*, *P.* *putida,* and *H. pylori* [21,41,55,56,57]. In principle, two square coverslips are placed on each side of a slide, around 16 mm apart. Agarose plugs are prepared in the middle of the two coverslips by pipetting 5–12 μL of preheated low melting point agarose (LMA), containing the effector to be tested or only PBS as control. Immediately, a third glass coverslip is placed over the bridge, using the edge of the other two coverslips as a stand. The overnight cells are then pipetted between the microscope slide and third glass coverslip and observed by microscopy and photographs are taken of the area at the edges of the plugs after 5–30 min where the chemotactic bands (density of cells) form around the agarose plug. This method is semi-quantitative, aimed at testing attractants and requires skill in assembly of the in-plug bridge. While not used to assess campylobacteria, this method was employed to assess the chemotactic behaviour of *H. salinarum* [55] and demonstrated the cell migration toward glutamate.

A commercially available system based on a similar principle, μ-Slide from Ibidi (GmbH, Munich, Germany), had been tested for assessment of *C. jejuni* chemotaxis. This system allows the user to track the migration of bacterial cells microscopically and to quantitate the cell numbers by viable count. µ-Slide has two separate opposing reservoirs (60 µL in each), divided by a 1-mm narrow liquid transition zone with a volume of 10 µL. Chemotactic motility is measured in response to a chemoeffector gradient, formed between the two chambers, one containing the effector and the other containing bacterial cells in PBS. An LMA in PBS (pH 7.5) is applied to the transition zone. The first reservoir is then completely filled with the bacterial cell suspension in 1% LMA. The second reservoir is filled with 60 µL of chemoeffector (final concentration of 10 mM) mixed with 0.7% of LMA, to final concentration of 0.35% and incubated for 3 h. After that, a 20 µL of sample from the chemoeffector reservoir is taken and enumerated by viable count. The μ-Slide chemotaxis system used campylobacters and allowed detection of the chemoresponses to attractants or repellents, it also enabled the tracking of individual cells to study motility patterns, such as in *C. jejuni* NCTC11168-O [38,39]. The non-motile strain (*flaAB*) and non-chemotactic strain (*cheY*) were used to validate the assay. This assay is easy to use but requires freshly prepared and active *C. jejuni* cells, a humid environment to avoid the drying of the agarose over the incubation period, and minimum movement of the slide during manipulation for accurate and reproducible results.

## 5. Comparison of t-HAP, Nutrient-Depletion and μ-Slide Assays

**Figure 1:**
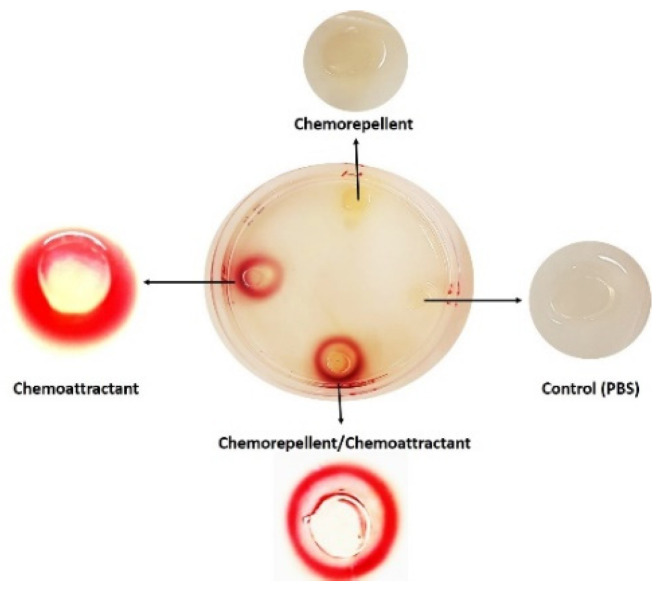
Schematic representation of the tHAP assay showing how t-HAP assesses both the positive and negative chemotactic responses of *C. jejuni*. The chemoresponse of the wild-type *C. jejuni* after 3 h incubation under microaerobic conditions at 42 °C with 100 mM of chemoattractant (serine), chemorepellent (arginine), and a mix of chemoattractant/chemorepellent (serine/arginine), from [26].

Nutrient-depletion assay, t-HAP and μ-slide chemotaxis appear to offer the most advantages for assessing both chemoattractant and chemorepellent responses. Here, we compare quantitative data from previously published t-HAP, nutrient-depletion and μ-slide assays [26,41] for measurements of the chemotactic motility of *C. jejuni* 11168-O, and its Δ*tlp10^LBD^* isogenic mutant strain [39]. All three assays were in agreement in establishing the repertoire of chemoattractants and chemorepellents for Tlp10. The comparison of the quantitative response measurements, using fucose, isoleucine, and aspartate as examples, showed a significant reduction in the chemoresponses to *C. jejuni* 11168-O Δ*tlp10^LBD^* isogenic mutant as compared to the wild type strain, which indicates that all three ligands are attractants (Table 2). Table 2 shows that the nutrient-depletion assay is more sensitive in terms of quantitation, as the reduction of chemoresponses to fucose was 1.98 for the Δ*tlp10^LBD^* mutant vs. *C. jejuni* 11168-O wild type strain, as compared with the t-HAP assay that registered a 1.4 log reduction for the mutant strain. Similarly, chemotaxis of the Δ*tlp10^LBD^* mutant toward isoleucine and aspartate was reduced by 1.7 and 2.29 log, respectively, in the nutrient-depletion assay and only 0.76 and 1 log when using t-HAP. The reduction in the level of chemoresponses indicates that the nutrient-depletion assay is more sensitive in the detection of the response differences between *C. jejuni* 11168-O wild type and mutant strains as compared to the t-HAP assay. However, t-HAP offers the advantage of providing a visual assessment of the chemoresponses toward a chemoattractant or a chemorepellent (Figure 1), and still allows quantitative analysis via the viable count.

**Figure 2:**
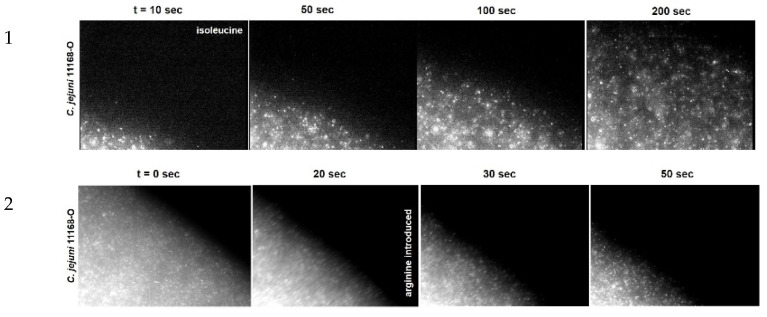
Time-lapse imaging of fluorescently labelled migrating *C. jejuni* 11168-O cells (from [39]). (**1**) Attractant response of WT (11168-O) toward 10 mM isoleucine. (**2**) Repellent response of WT (11168-O) cells partially migrated toward 10 mM isoleucine from the left chamber of μ-Slide, followed by the introduction of 10 mM arginine into the right chamber at 20 s. Scale bar = 100 µm.

The μ-slide chemotaxis assay showed promising results, where the assay enabled both a compelling and recordable visual observation and similar quantification of the chemoresponses of *C. jejuni* [38,39] (Figure 2). Figure 2 (extract from [39]) shows the cell migration of wild-type *C. jejuni* strains 11168-O (11168-O WT) toward an attractant (isoleucine) and away from a repellent (arginine).

Overall, the t-HAP assay is quick and easy and allows comparison of different concentrations of chemoeffectors in a short time. However, both the nutrient-depletion assay and the μ-slide assay are more suitable for studying chemotactic motility at biologically relevant concentrations.

## 6. Conclusions and Recommendations

Here we discussed several canonical chemotaxis assays used for the study of the *C. jejuni* chemotaxis and considered the modifications made to these methods to retain their advantages and circumvent the limitations. Each assay addresses the fundamental question of “how do bacteria respond to chemical stimuli”? However, while some methods are easy to establish and perform, they suffer from excessive variability or lack of the ability to quantitate chemotactic responses. Other assays require highly trained personnel and specialized instrument or are laborious, lack reproducibility and consistency. Therefore, the choice of a method should be informed by the aim of the study and previously accumulated knowledge. Whatever method is chosen, it is critical to include appropriate chemotaxis-specific positive and negative controls, to consider the pH of the solutions to exclude taxis away from the acidic or basic environments, to consider the role the effector may play in the biology of the organism, and to ensure that the concentration of the effector is within a biologically relevant range. The combination of several assays is also commendable to enhance the confidence in the veracity of the results.

## Figures and Tables

**Table 1:** Advantages and disadvantages of common chemotaxis assays. M- Molar, mM- Millimolar.

Method	Detection Time	Molar Concentration	Advantages	Disadvantages	References
Agar-based assays					
Hard-plug agar assay (HAP assay)	3 h	10–100 mM	-Easy to prepare. -Gives quantitative data. -Requires minimal equipment. -Strains can be compared directly.	-Chemorepellent taxis are difficult to observe. -False positive results are possible.	[16]
Modified hard-plug agar assay (t-HAP assay)	10 min to 3 h	10–100 mM	-Easy to prepare. -Gives quantitative data. -Requires minimal equipment. -Strains can be compared directly. -Differentiations between catabolised and non-catabolised ligands are possible	-Chemorepellent taxis are difficult to observe.	[26]
Nutrient-depletion assay	3–6 h	2–10 mM	-Gives quantitative data. -Easy to prepare. -Requires minimal equipment. -Strains can be compared directly. - chemorepellents taxis can be quantitated. -Gradients are created by diffusion, not metabolism.	-Sensitive to any motions around the assays. -One strain and conditions can be monitored per assay. -Visual observation is difficult.	[34,35]
Tube-based assay	75 h	1 M	-Easy to prepare. -Requires minimal equipment. -Strains can be compared directly.	-Not suitable for studying chemorepellents. -Semi-quantitative.	[36]
Capillary assay					
Capillary assay	1 h	10–100 mM	-Gives quantitative data. -Requires minimal equipment. -Gradients are created by diffusion, not metabolism.	-Not suitable for studying chemorepellents. -One strain and condition can be monitored per assay.	[37]
Chemotaxis chamber					
μ-slide chemotaxis chamber	3 h	5–10 mM	-Ideal to study the behaviour of a single cell. -Chemoresponses can be measured for a group of cells or a single cell. Clear visualisation of cell migration. -Gives quantitative data.	-One strain and condition can be monitored per assay. -Tracking system is relatively expensive.	[38,39]

**Table 2:** Chemotaxis assays of wild-type *C. jejuni* strains 11168-O (11168-O WT), and Tlp10^LBD^ mutant (Δ*tlp10*) (from [39]). The viable count (Log_10_ CFU/mL) differences between t-HAP, Nutrient-depletion and μ-slide assays of 11168-O WT, Δ*tlp10*.

Ligands	t-HAP		Nutrient-Depletion Assay		μ-Slide Assays	
	11168-O WT	Δ*tlp10*	11168-O WT	Δ*tlp10*	11168-O WT	Δ*tlp10*
fucose	6.7 ± 0.61	5.3 ± 0.02	5.8 ± 0.63	3.86 ± 0.43	4.96 ± 0.39	3.61 ± 0.08
isoleucine	6.6 ± 0.34	5.9 ± 0.18	5.7 ± 0.4	4 ± 0.61	6.61 ± 0.33	5.52 ± 0.17
aspartate	6.38 ± 0.4	5.33 ± 0.22	5.61 ± 0.93	3.32 ± 0.18	5.71± 0.4	4.21 ± 0.16

## Data Availability

Not applicable.
